# Fortification of Durum Wheat Pasta with Nut Oil Cake: Effects on Nutritional and Technological Properties

**DOI:** 10.3390/molecules30163411

**Published:** 2025-08-18

**Authors:** Dorota Gałkowska, Karolina Pycia, Anastasiia Krykhta

**Affiliations:** 1 Department of Food Analysis and Evaluation of Food Quality, University of Agriculture in Krakow, Balicka 122, 30-149 Krakow, Poland; dorota.galkowska@urk.edu.pl (D.G.); anastasiia.krykhta@student.urk.edu.pl (A.K.); 2 Department of Food Technology and Human Nutrition, Institute of Food Technology and Nutrition, Faculty of Technology and Life Sciences, University of Rzeszów, Zelwerowicza 4, 35-601 Rzeszów, Poland

**Keywords:** pasta, hazelnut, walnut, nut oil cake, antioxidant properties, texture

## Abstract

The study aimed to produce semolina pasta enriched with walnut or hazelnut oil cake and to investigate its nutritional and technological properties. The pasta was prepared by substituting 10% of semolina with walnut or hazelnut oil cakes. The chemical composition, antioxidant properties, and culinary characteristics of the pasta were determined. Additionally, the texture and color of uncooked and cooked pasta were examined using instrumental techniques. The enriched pastas showed higher protein, fat, ash, and dietary fiber contents compared to standard pasta (SP). Walnut oil cake pasta (WOCP) had the highest protein content, amounting to 15.8 g/100 g dry weight (d.w.), while hazelnut oil cake pasta (HOCP) had the highest dietary fiber content (6.75 g/100 g d.w.). Moreover, the enriched pastas showed significantly higher antioxidant potential and total phenolic content, both before and after cooking. The total phenolic content (TPC) of cooked pasta ranged from 88.85 mg GAE/100 g d.w. (SP) to 145.48 mg GAE/100 g d.w. (WOCP). Compared to SP, the developed pastas required cooking times of 2–3 min longer and showed higher water absorption, accompanied by increased cooking losses. They were characterized by a specific, dark color and showed reduced hardness and lower elasticity after cooking compared to SP. Pasting properties further suggested that starch swelling was restricted by the nut oil cakes. Overall, incorporating walnut and hazelnut oil cakes enhanced the nutritional profile and imparted notable health-promoting attributes to the pasta, underscoring the potential of these by-products as functional ingredients in pasta formulations.

## 1. Introduction

The most popular cereal products include bread and pasta. Pasta offers many advantages, including ease of preparation, optimal nutritional value, attractive sensory features, suitability for long-term storage in dry form, and low price [1]. This product constitutes the basis for preparing many dishes, including regional specialties. Pasta dough is made by mixing durum wheat flour, called semolina (*Triticum turgidum* var. durum Desf.), with water [1,2]. Such a dough, with a water content of 28–32%, *w*/*w*, is kneaded mechanically, and then various forms of pasta are shaped and dried [1]. Semolina produced from durum wheat grain is the most suitable flour for high-quality pasta, distinguished by a very high protein content (10.9–13.5%), yellow color due to carotenoid pigments, and coarse granulation [1,2,3]. These features give cooked pasta an attractive yellow color and desirable taste characteristics. The nutritional value of pasta is primarily associated with high carbohydrate and protein contents. However, pasta made from refined durum wheat flour is characterized by a low content of bioactive substances such as vitamins, phenolic acids, flavonoids, and other biologically active compounds [2]. From the consumer’s perspective, it is reasonable to develop foods that, in addition to satisfying hunger, also have beneficial effects on health. Consequently, there has been growing interest in developing and assessing the quality of pasta enriched with valuable plant additives. In particular, the trend of using food industry by-products as ingredients to improve the chemical composition of pasta and to provide health-promoting properties is noteworthy. The latter aspect is related to the presence of phytochemicals in plant raw materials, mainly phenolic substances and carotenoids, which have a beneficial role in preventing cancer, metabolic, and cardiological diseases [2,4]. Epidemiological studies have shown that consuming food rich in phenolic compounds, which possess antioxidant properties, helps prevent many diet-related diseases [5].

Due to the global population growth and changes in consumers’ dietary habits, the demand for food continues to increase, leading to higher food production. This, in turn, results in the generation of large quantities of by-products from agricultural and food industries. These by-products include bran, husks, pomace, oil cake, etc., produced at various stages of food processing. Collecting these by-products is often problematic and costly; in many cases, producers are required to dispose of them. However, agri-food industry by-products are a valuable and inexpensive source of protein, dietary fiber, minerals, vitamins, and health-promoting bioactive substances. Polyphenols, in particular, are dominant among bioactive compounds, which have documented health-promoting properties such as antioxidant, cardioprotective, and anticancer effects. Reusing these by-products in food processing, e.g., as enrichment ingredients, makes it possible to improve food nutritional value while reducing negative environmental impact [6,7,8]. Notable examples of by-products are nut oil cakes (or meals). They are residues obtained after pressing or extraction of oil from nut kernels. Oil cakes are rich in protein, minerals, and valuable bioactive substances [9,10,11]. The literature on the subject includes numerous studies on developing pasta enriched with by-products of agri-food processing. For instance, grape pomace and seeds [12,13], powdered tomato peel [14], non-extruded and extruded blackcurrant pomace [15], linseed cake [16], olive cake [17], powdered leaves of *Cistus incanus* L [18], *Moringa oleifera* L. leaf powder [19], dried parsley leaves [20], and the dried powdered leaves of wild garlic (*Allium ursinum*) [21] have been used. The enriched pasta products developed by the cited authors were characterized by increased nutritional value and desired functional properties. Covaliov et al. [22] found that enriching pasta with pumpkin seed, sunflower seed, and walnut flour improved its amino acid profile, especially lysine content, which is often deficient in wheat products. Given these findings, it is of interest to investigate the effect of incorporating nut oil cakes into wheat pasta on its functional properties and nutritional value.

Therefore, the aim of this study was to produce semolina pasta enriched with walnut and hazelnut oil cakes and to investigate its technological and nutritional characteristics.

## 2. Results and Discussions

### 2.1. Chemical Composition of Raw Materials and Products

The protein content in semolina was 13.0% (d.w.) (Table 1), characteristic of durum wheat milling products [23]. In pasta made solely from semolina (SP), the protein content corresponded to that of the raw material. WOC contained nearly three times more protein than semolina, while HOC contained approximately 2.5 times more. The literature reports that the protein content in walnut oil cakes varies widely, ranging from 10.30% [24] through 30.45% [25] and up to 50.4% [26]. In our study, the protein content of HOC was almost identical to that reported by Lučan Čolić et al. [27], but much lower than 45.6% reported by Gul et al. [28]. Substituting 10% of the amount of semolina in the pasta recipe with HOC, which is a better protein source than semolina, increased the protein content in the final product (Table 1).

Both WOC and HOC were characterized by very high fat content compared to semolina. According to the literature data, fat levels in walnut oil cakes can range from 17.3% to 55.7% (d.w.) [24,25,26,29], and in hazelnut oil cakes, from 8.41% to 27.9% (d.w.) [26,27,30]. The variation depends on nut variety and the method of obtaining the oil cake. The presence of WOC or HOC in the pasta composition resulted in a significant (*p* < 0.05) increase in the fat content in the cooked pasta, more than two and a half times, as compared to SP.

Mineral content, determined as ash, was significantly (*p* < 0.05) higher in WOC and HOC than in semolina (Table 1), consistent with the literature data. Walnut oil cakes studied by Niyonshuti and Kırkpınar [31] and hazelnut oil cakes analyzed by Ozdemir et al. [30] contained, respectively, 4.8% and 6.05% of minerals in dry mass. Cooked pasta enriched with the oil nut oil cake (WOCP and HOCP) was characterized by an increased content of minerals compared to SP. However, when estimating the mineral content of the uncooked pasta, the cooking process contributed to its significant (ca. 50%) reduction.

In our study, the nut oil cakes were characterized by high total dietary fiber (TDF) content, which was more than five times and more than eight times higher than in semolina, respectively, for WOC and HOC (Table 1). Walnut oil cakes are known to be good sources of dietary fiber, especially soluble fraction (SDF) [32,33]. The total dietary fiber content of WOC determined by us significantly exceeded the values reported by other authors, 7.2% (d.w.) [24] and 9.4% (d.w.) [29], while HOC’s TDF was similar to that reported by Panzanini et al. [33] (25.02–26.33%, d.w.). Cooked WOCP and HOCP had higher insoluble fiber (IDF) content than cooked SP. However, that cooked pasta had a lower SDF level in relation to the amount contributed by the raw ingredients. This was most likely due to losses during cooking or the transformation of part of the soluble fiber fraction into the insoluble one. Ultimately, these changes resulted in only HOCP having a significantly (*p* < 0.05) higher TDF content than SP (Table 1). In accordance with the European Union guidelines on nutrition and health claims used in the labelling, presentation, and advertising of food [34], our pasta with hazelnut oil cakes can be considered a high dietary fiber foodstuff.

In contrast to semolina, WOC and HOC had significantly lower digestible carbohydrate contents (Table 1). Montrimaitė and Moščenkova [26] showed that the walnut oil cakes and hazelnut oil cakes they studied contained, respectively, 22.44% (d.w.) and 29.92% (d.w.) of carbohydrates, including dietary fiber. In turn, Lučan Čolić et al. [27] showed almost 34% (d.w.) of carbohydrates, including dietary fiber, in cold-pressed hazelnut oil cake. The nut oil cakes we examined were, therefore, characterized by a higher content of total carbohydrates than the nut oil cakes analyzed by the authors cited above. As expected, substituting some of the semolina in the pasta recipe with nut oil cakes resulted in a decrease in digestible carbohydrate levels in the cooked pastas, but they remained excellent sources of digestible carbohydrates (approx. 74–75%, d.w.).

### 2.2. Antioxidant Potential and Total Phenolic Content

Table 2 presents antioxidant potential and total phenolic content of uncooked and cooked pasta. In the case of uncooked pasta, the pasta enriched with walnut oil cakes (WOCP) exhibited significantly higher antioxidant activity compared to standard pasta (SP), indicating the potential of the oil nut cakes to impart health-promoting properties to semolina pasta. The observed properties of the enriched pasta resulted from a significantly higher, by over 75%, content of polyphenolic compounds as compared to SP (Table 2). The presence of hazelnut oil cakes in the pasta recipe did not cause a significant change in the antioxidant activity of the final product measured by ABTS and FRAP assays. At the same time, a lower content of phenolic compounds was determined in HOCP as compared to SP.

The changes in the antioxidant potential of uncooked pasta as affected by the oil nut cakes were similar to the changes in this parameter of cooked pasta. WOCP was characterized by a significantly higher antioxidant potential than SP (Table 2). Moreover, the TPC of WOCP exceeded that of SP by over 60%. Pasta with HOC did not differ significantly from SP in terms of either antioxidant activity determined by ABTS assay or reducing capacity (FRAP assay).

The increase in both the antioxidant potential and the total polyphenol content of wheat pasta as a result of enriching it with plant or animal raw materials has already been described in the scientific literature [2]. Simonato et al. [17] showed that fortification of pasta with olive pomace increased its antioxidant potential and total polyphenol content. However, there are few data in the literature on the antioxidant potential of walnut and hazelnut oil cakes, or information on the amount and profile of polyphenols in these raw materials. A few researchers reported that walnut flour cakes [35] and hazelnut oil cakes [36] were good sources of natural antioxidants, especially phenolic compounds. It is known, however, that walnuts and hazelnuts are abundant in phenolic compounds and are characterized by very high antioxidant potential [37,38]. In the case of walnuts, analysis of the polyphenol profile by ultra-performance liquid chromatography coupled with a mass detector (UPLC-PDA-ESI-MS) technique allowed the identification of mainly phenolic acids, including gallic acid, procatechinic acid, syringic acid, and genistinic acid [37]. In turn, compounds with strong antioxidant potential, such as gallic acid, chlorogenic acid, catechins, and kaempferol-3-O-hexoxyl-hexoside, were identified in dry hazelnuts [38]. Pycia et al. [39] showed a several-fold increase in the antioxidant potential and total phenolic content of wheat breads fortified with laboratory walnut oil cakes as compared to a control bread. Moreover, the results of the polyphenol profile analysis showed that caffeic, ferulic, sinapic, and coumaric acids were present in the breads enriched with walnut oil cakes, and their content increased significantly with the increasing share of the cakes in the bread recipe. Wójcik et al. [35] and Pop et al. [24] used walnut flour/walnut oil cakes powder as an ingredient of low-carbohydrate bread and macarons, respectively. In turn, Bursa et al. [36] used hazelnut oil cake as a partial replacer of sugar and milk-originated powders in chocolate. In the cases cited, the authors showed an increase in the antioxidant potential or total polyphenol content of products containing the above-mentioned by-products. The above reports prove that due to the presence of bioactive substances and strong antioxidant potential, walnut oil cake still constitutes a valuable by-product of nut processing.

In line with the uncooked pastas, the antioxidant potential of cooked WOCP remained the highest among the analyzed cooked pastas (Table 2). The polyphenol content in walnut oil cakes (WOC) and hazelnut oil cakes (HOC) was 1685 mg GAE/100 g dry weight and 170 mg GAE/100 g dry weight, respectively, which is reflected in the polyphenol contents of the pasta. The TPC of WOCP was also over 1.6 times higher than that of SP. HOCP’s phenolic content and antioxidant activity did not differ significantly from the values of these parameters determined in the SP. It is worth noting that the TPC of SP and HOCP increased by almost 10% and 2%, respectively, after cooking. This suggests cooking may release phenolics from macromolecular compounds [40]. In turn, the lack of considerable increase in the TPC of WOCP as affected by cooking might have resulted from the coexistence of the above-described phenomenon and degradation of phenolic compounds under the influence of high cooking temperature [41,42]. Simonato et al. [17] studied the antioxidant potential and total polyphenol content of olive pomace enriched pasta and observed that cooking pasta reduced its phenolic content by 70%. According to the cited authors, the phenolic fraction of olive pomace contains derivatives of tyrosol, luteolin, oleuropein, and quercetin, compounds that are more soluble in water than conjugated forms requiring alkaline or acid hydrolysis before extraction.

### 2.3. Cooking Properties of Pasta

Table 3 shows the results of determination of culinary properties of standard pasta and pasta enriched with the nut oil cakes. The optimal cooking time of the enriched pasta was extended by 2 min or 3 min, respectively, for HOCP and WOCP, as compared to the optimal cooking time of SP. Longer cooking time may indicate hindered water penetration into the pasta and, as a consequence, slower swelling of the starch grains [43]. The reason for this phenomenon could be the different structure of the pasta, which could be influenced by the following factors. The first one was higher fat content of the pasta; the lipids could have coated the starch grains and consequently hindered their swelling. The second factor could have been the interactions of the semolina components with the nut oil cakes’ components, leading to a tighter, more compact structure of the pasta, constituting a physical barrier for water penetrating the interior of the pasta. A reduction in the cooking time of wheat pasta as a result of enriching it with food processing by-products was reported, among others, by Gałkowska et al. [15], Gull et al. [5], Padalino et al. [14], and Simonato et al. [17], who used, respectively, blackcurrant pomace, millet flour-carrot pomace mix, tomato peel flour, and olive pomace. The cited authors indicated that the shortening of the pasta cooking time could have resulted, on the one hand, from the reduced share of gluten proteins in the product composition and, consequently, from the weakened starch-protein structure, and on the other hand, from the presence of dietary fiber and, as a result, the formation of a looser pasta structure, which facilitated water penetration and accelerated starch gelatinization.

Water absorption is a parameter that informs about the amount of water that the pasta retains after cooking [5]. It was shown that the pasta fortification with HOC significantly (*p* < 0.05) increased water absorption (Table 3), while WOCP’s water absorption was similar to that of SP. Simonato et al. [17] and Lončarić et al. [44] also observed an increase in the water absorption of pasta due to its fortification with, respectively, olive pomace and powdered apple peel, and showed that the value of this parameter increased with the increasing share of the plant additive. Similar to the optimal cooking time, the observed trend may have been due to the higher amount of fiber in the enriched pasta. This component, by weakening the structure of the pasta, facilitates the penetration of water into the internal structure and its absorption.

The swelling index shows the amount of water absorbed by both starch and proteins during the cooking process, necessary for starch gelatinization and protein hydration [5]. In line with the trend observed for water absorption, the fortified pasta was characterized by higher swelling index values compared to SP (Table 3). It can be assumed that the higher protein content of WOCP and HOCP compared to SP (Table 1) contributed to binding more water than in the case of SP. It was also found that HOCP was characterized by a higher swelling index than WOCP, which can be attributed to higher soluble fiber content of HOCP. Simonato et al. [17] reported that the replacement of durum wheat semolina with 5 or 10% of olive pomace increased the swelling index of the resulting pasta. In turn, in the study by Gałkowska et al. [15], the substitution of 5 or 10% of blackcurrant pomace for durum wheat semolina resulted in a decreased swelling index of the fortified pasta.

Another parameter used to predict the quality of pasta is cooking loss. During pasta cooking, soluble parts of starch and other soluble components leach into the water and, as a result, the cooking water becomes cloudy [41]. Cooking loss depends on pasta composition, especially on the quantity and quality of gluten and degree of starch damage, as well as on pasta drying method [45]. For the pasta studied, values of the cooking loss achieved acceptable level, since according to pasta industry guidelines, the cooking loss should not exceed 8% of the dry weight [46]. Fortification of pasta with the nut oil cakes resulted in a significant (*p* < 0.05) increase in the value of the parameter discussed (Table 3). This may be related to both a slightly longer cooking time of the enriched pastas as compared to SP, as well as to a different qualitative composition. Compounds deriving from walnut or hazelnut oil cake, such as fiber and polyphenols, could also be lost in water [17]. Tudoriča et al. [47] reported increased cooking losses for dietary fiber enriched pasta samples. This phenomenon was explained by a disruption of the starch–protein network and the uneven distribution of water within the pasta matrix resulting from the competitive hydration tendency of the fiber, resulting in reduced starch swelling. Conversely, a decrease in the cooking losses of pasta enriched with flaxseed cake or flaxseed flour as compared to the control pasta was observed by Zarzycki et al. [16]. According to the cited authors, lignans contained in flaxseeds due to high water absorption ability increase viscosity and, in consequence, limit the migration of dry matter components, e.g., starch, during the cooking of pasta.

### 2.4. Color Parameters of Pasta

**Figure 1:**
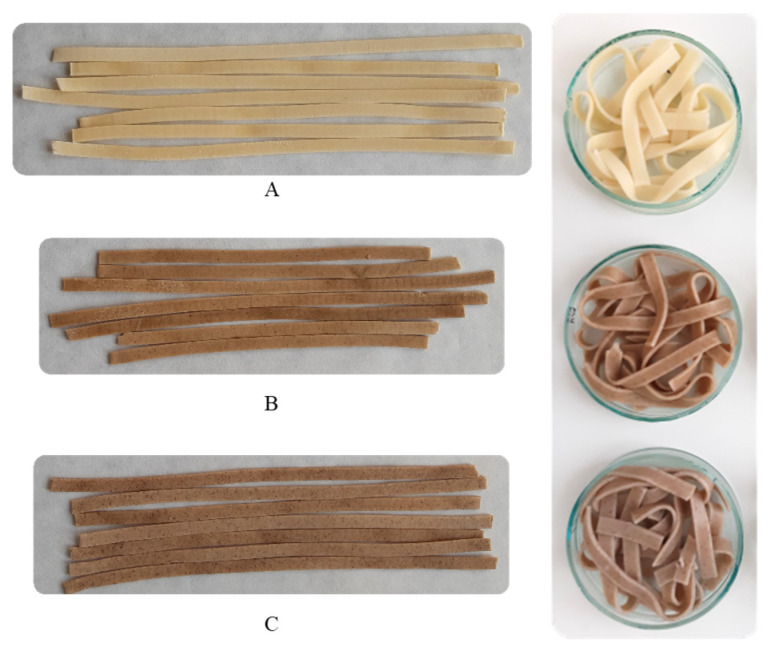
Image of pasta samples. (**A**): standard pasta (SP); (**B**): pasta with walnut oil cake (WOCP); (**C**): pasta with hazelnut oil cake (HOCP). (**Left**): uncooked pasta. (**Right**): cooked pasta.

Color is one of the most important quality parameters considered by the consumer when purchasing a food product. Table 4 summarizes the color parameters of uncooked and cooked pasta, while Figure 1 shows the appearance of the samples before and after cooking.

In general, durum wheat pasta is naturally yellow due to the presence of carotenes and xanthophylls in the raw material [16]. The membranous shell of walnut and hazelnut seeds contains natural carotenoid pigments such as lutein, zeaxanthin, and β-carotene [48,49,50]. The above dyes should, therefore, be expected in the cakes remaining from pressing oil from walnut and hazelnut kernels. WOCP and HOCP had a characteristic color derived from the color of the enriching ingredients. The color of enriched pasta, both uncooked and cooked, was visibly darker than the color of SP, which was reflected in significantly lower (*p* < 0.05) L* values (Table 4). WOCP and HOCP did not differ significantly in color lightness. A significant decrease in the color lightness (L*) of durum wheat semolina pasta due to the introduction of olive pomace or flaxseed oil cake to its recipe was found in studies by Simonato et al. [17] and Zarzycki et al. [16], respectively. The color of WOCP, both before and after cooking, was characterized by increased shares of red and yellow hues compared to the color of SP. In turn, the color of HOCP was characterized by a higher share of red hues but a lower share of yellow hues than SP. Heat treatment resulted in a decrease in the values of a* and b* parameters of all the analyzed pasta, likely due to the partial loss of carotenoid pigments from both semolina and nut oil cakes.

### 2.5. Texture Parameters of Pasta

Uncooked pasta was subjected to a three-point bending test to determine the bending force, a measure of hardness, and displacement at fracture. SP turned out to be harder than WOCP and HOCP (Table 5).

As a result of introducing WOC or HOC into the pasta recipe, its hardness was reduced by 40% and over 52%, respectively. The lower breaking force of the enriched pasta was accompanied by a smaller displacement of the sampler leading to sample breakage, while the difference between the values of this parameter obtained for WOCP and HOCP was statistically insignificant. It can be assumed that the reduced mechanical strength of pasta containing the nut oil cakes was mainly due to an approximately three times higher fat content in these pastas compared to SP (Table 1). As reported by Gałkowska et al. [15], replacing 10% of the semolina mass with non-extruded or extruded blackcurrant pomace powder had a different effect on the hardness of uncooked pasta.

The results of the tensile test of cooked pasta showed that the values of elongation at break were significantly (*p* < 0.05) reduced in WOCP and HOCP compared to SP (Table 5). However, it was found that both the breaking force and tensile strength of WOCP did not differ significantly (*p* < 0.05) from SP, whereas HOCP showed lower values of these parameters than those determined for SP. This means that the enriched pasta in question was characterized by lower elasticity and firmness than the standard pasta. These changes in physical characteristics may result from significantly higher water absorption and greater cooking loss of HOCP as compared to SP (Table 3). The reason for this may be the higher dietary fiber content of the pasta before cooking (Table 1). This ingredient, with high affinity for water, most likely hindered the access of sufficient water required to develop the gluten network in the pasta dough. This resulted in the formation of a weaker, discontinuous protein structure within which the starch granules were trapped. As a consequence, starch granules swelling during pasta cooking could destroy the protein network, resulting in soft textured pasta [51,52]. A decrease in the tensile strength and elongation rate of durum wheat pasta fortified with 6–15% of turmeric residue powder was reported by Long et al. [53]. Similarly, 10% substitution of durum wheat semolina with watermelon rind powder in pasta formulation resulted in a product of significantly reduced elongation rate and tensile strength [54]. According to Shiau et al. [55], excessive dietary fiber, especially its insoluble fraction, has a negative effect on the structure of the protein–starch matrix, owing to fiber–gluten interaction and the dilution of wheat gluten of pasta.

### 2.6. RVA Parameters of Pasta Powder

**Figure 2:**
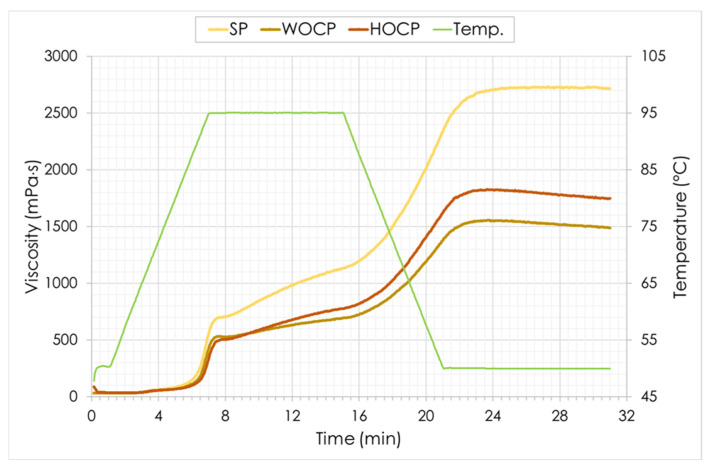
Pasting profile of pasta powders. SP—standard pasta; WOCP—pasta with walnut oil cake; HOCP—pasta with hazelnut oil cake; Temp.—temperature.

The tested pastas showed a specific gelatinization characteristic, expressed by the absence of a clear viscosity peak during the heating phase (Figure 2). Such a sample behavior at pasting test suggests that starch grains were trapped inside a matrix that limited their swelling and subsequent gelatinization and dissolution in an aqueous system [56]. The enrichment of durum wheat flour pasta with the nut oil cakes significantly influenced the change in the viscosity characteristics of pasta powders compared to SP (Table 6; Figure 2).

The gelatinization process of the starch contained in WOCP and HOCP began at higher temperatures than in the case of SP. This phenomenon can be interpreted in the context of the higher temperatures required to cook the enriched pasta [57]. Moreover, the viscosity of the pastes formed by WOCP and HOCP powders turned out to be lower than the viscosity of the system formed by SP at each stage of the test. These observations may suggest that starch swelling was significantly limited in the presence of components derived from the nut oil cakes. Moreover, possible interactions of starch and proteins of semolina with the components of nut cakes, especially lipids, occurring during the formation of pasta dough and its subsequent drying could have resulted in the formation of a compact structure with lower water absorption and volume expansion. Tazrart et al. [58], examining dried pasta in which 10% of semolina was replaced with broad bean flour, showed that the enriched pasta produced a system characterized by lower peak viscosity and final viscosity values than the control pasta. These changes were attributed to the dilution and weakening of the gluten matrix.

## 3. Materials and Methods

### 3.1. Materials

Re-milled durum wheat semolina (Molino F.Lli CHIAVAZZA S.p.A., Casalgrasso, Italy) was bought in a local supermarket. Walnut oil cake (WOC) and hazelnut oil cake (HOC) were kindly supplied by the Warmińska Manufaktura (Jeziorany, Poland). The oil cakes were in powdered form, which was sieved through a sieve with a mesh size of 850 µm.

### 3.2. Methods

#### 3.2.1. Pasta Preparation

Three pasta formulations were prepared as follows. The semolina or a mixture of the semolina and walnut oil cake or hazelnut oil cake, where 10% by weight of the amount of semolina was substituted with the walnut oil cake or hazelnut oil cake, was mixed with distilled water at the weight ratio of 2.5:1 using a SPM-3 pasta maker (Semco Corporation, Shenzhen, China). The resulting pasta dough was passed through a lab-scale pasta machine (Atlas 150, Marcato S.p.A, Campodarsego, Italy). Pasta ribbons of approx. 1 mm thick, 5 mm width, and 200 mm length were shaped and dried in a laboratory incubator (Climacell 222, BMT Medical Technology s.r.o., Brno, Czech Republic) following the drying procedure described by Gałkowska et al. [59]. The following symbols were adopted to describe the samples: SP—standard pasta (made only from semolina); WOCP—pasta with walnut oil cake; HOCP—pasta with hazelnut oil cake.

#### 3.2.2. Determination of Chemical Composition of Raw Materials and Products

The chemical composition of the pasta was determined after grinding in a knife mill (GM 200; Retsch GmbH, Haan, Germany). Water content was determined by drying at 130 °C for 1.5 h in a forced air oven. Protein content was assessed by the Kjeldahl procedure (using a nitrogen-to-protein conversion factors of 5.7 and 5.3 for semolina and walnut or hazelnut oil cake, respectively, considering their mass fraction in the pasta) after sample digestion in concentrated sulphuric acid. Fat content was determined by hydrolysis in 4 M hydrochloric acid and subsequent Soxhlet extraction with petroleum ether. Ash content was determined by incineration in a muffle furnace at 900 °C for 4 h. Total (TDF), soluble (SDF), and insoluble (IDF) dietary fiber contents were determined by the enzymatic-gravimetric method using a total dietary fiber kit (Megazyme Ltd., Bray, Ireland) in accordance with the AOAC 991.43 standard [60]. Available carbohydrate content was calculated according to the difference method (100 % − (moisture % + protein % + fat % + ash % + fiber %)). All determinations were carried out in triplicate. The results were expressed in g per 100 g of dry weight (d.w.).

#### 3.2.3. Determination of Antioxidant Potential and Total Phenolic Content

##### Sample Preparation

In order to determine the antioxidant potential and total phenolic content of uncooked and cooked pasta samples, methanol extracts of the samples were prepared. Before extraction, uncooked pasta was ground to powder in an electric mill (TSM6A013B, Bosch, Gerlingen-Schillerhöhe, Germany), while cooked pasta was prepared as follows. Pasta was cooked for the designated time (as described in Section 3.2.4), then cooled, frozen, dried in a freeze dryer (Alpha 1–2 LDplus, Donserv, Warsaw, Poland), and ground to a fine powder in an electric mill (TSM6A013B, Bosch, Gerlingen-Schillerhöhe, Germany). For extraction, 1 g of powder was mixed with 10 mL of 80% aqueous methanol and sonicated at 30 °C for 30 min in an ultrasonic cleaner (Sonic 10, Polsonic, Warsaw, Poland). The mixture was centrifuged at 7500 rpm, and the supernatant was decanted for analysis [61]. The procedure of extraction was performed in triplicate.

##### Determination of Antioxidant Properties

The antioxidant properties of pasta were measured by three spectrophotometric methods: the 2,2′-azino-bis-3-ethylbenzthiazoline-6-sulphonic acid (ABTS) assay [62], the 1,1-diphenyl-2-picrylhydrazyl (DPPH) assay [63], and the ferric ion reducing antioxidant potential (FRAP) assay [64]. Measurements were performed using a UV-Vis spectrophotometer (Nicolet Evolution 300 Type U-2900, HitachiThermo, Dallas, TX, USA). Briefly, in the case of the ABTS assay, an aliquot of 3 mL of ABTS radical cation solution (Sigma-Aldrich, Darmstadt, Germany) was added to 0.03 mL of appropriately diluted methanol extract (prepared as described in Section Sample Preparation). After 6 min, the absorbance was read at a wavelength of 734 nm against distilled water. In the DPPH assay, 0.5 mL of appropriately diluted methanol extract was mixed with 2 mL of DPPH radical solution (0.003 mg/100 mL) (Sigma-Aldrich, Darmstadt, Germany) and incubated for 10 min. After this time, the absorbance was read at a wavelength of 517 nm against methanol. The FRAP assay consisted in adding 3 mL of the freshly prepared FRAP reagent (Sigma-Aldrich, Darmstadt, Germany) to 0.5 mL of appropriately diluted extract. After 10 min, the absorbance was read at a wavelength of 593 nm against distilled water. The antioxidant activity of the tested pastas was expressed in millimoles of Trolox equivalents (TE) per 100 g, d.w. Analyses were performed in triplicate.

##### Determination of Total Phenolic Content

Total phenolic content (TPC) was determined using the Folin–Ciocâlteu assay [65]. Two mL of distilled water, 0.2 mL of Folin–Ciocâlteu reagent (Sigma-Aldrich, Darmstadt, Germany), and 1 mL of 20% (*w*/*v*) Na_2_CO_3_ solution (Chempur, Piekary Slaskie, Poland) were added to 0.1 mL of appropriately diluted extract. After mixing, the sample was kept in the dark for 1 h, then the absorbance was read at 765 nm against distilled water. The TPC was expressed in mg of gallic acid equivalents (GAE) per 100 g, d.w. Analyses were performed in triplicate.

#### 3.2.4. Determination of Cooking Properties of Pasta

The optimal cooking time of pasta was determined according to the official method [66]. Pasta samples were cooked in boiling distilled water, and a single pasta strand was compressed between two thin glass plates at 30 s intervals. The optimal cooking time corresponded to the time required for the disappearance of the ‘white core’ or ‘dark core’ of the semolina pasta strand or enriched pasta strand, respectively. Other culinary characteristics (water absorption, swelling index, and cooking loss) were determined as described by Gałkowska et al. [15]. The water absorption, expressed in grams of water absorbed by 100 g of raw pasta during cooking at predetermined optimum cooking time, was measured by weighing pasta before and after cooking. The swelling index, expressed in grams of water absorbed by 1 g of dry weight of pasta during cooking at predetermined optimum cooking time, was determined by weighing pasta before cooking and after drying at 105 °C to a constant weight. The cooking loss, expressed in grams of dry matter loss per 100 g of raw pasta, was measured by determining the weight of solids lost into the cooking water after drying at 105 °C to a constant weight. The procedures were performed in duplicate.

#### 3.2.5. Determination of Color Parameters of Pasta

The color of uncooked and cooked pasta was measured using a color i5 spectrophotometer (X-Rite Incorporated, Grand Rapids, MI, USA). The color parameters, L* (lightness value), a* (green/red), and b* (yellow/blue), were measured in the CIELab system, in SPEX mode, using D65 standard illuminant and 10° observer. The measurements were performed in five repetitions.

#### 3.2.6. Determination of Texture Parameters of Pasta

The pasta texture tests were performed using an EZ-LX Testing Instrument (Shimadzu Corporation, Tokyo, Japan). Uncooked pasta was tested in a three-point bending test using a distance between support of 30 mm and punch speed of 1 mm/s. Break force (N) and displacement at fracture (mm) were recorded. For the tensile test, the pasta was cooked for previously determined optimal cooking time, maintained for 1 min in cold distilled water, drained, and then left to rest for approximately 15 min in a closed plastic Petri dish. The test was performed with a roller type noodle tensile jig moving with a speed of 3 mm/s, where breaking force (N), elongation at break (%), and tensile strength (kPa) were determined. The measurements were performed in four repetitions.

#### 3.2.7. Determination of RVA Parameters of Pasta Powder

The pasting properties of pasta powder were determined with a Rapid Visco Analyzer (TecMaster, Perten Instruments, Macquarie Park, Australia).. Pasta samples were ground in a knife mill (GM 200; Retsch GmbH, Haan, Germany) and suspensions of 12.0% (*w*/*w*) in water were prepared. A 31 min test was performed according to the procedure of Bruneel et al. [51] and the following parameters were determined: pasting temperature (°C), peak viscosity (mPa·s), and final viscosity (mPa·s). The measurements were performed in triplicate.

#### 3.2.8. Statistical Analysis

The data were subjected to a one-way analysis of variance (ANOVA), and the means were compared using Tukey test at significance level of 0.05. Statistical analysis was performed using the Statistica 13.3 software (TIBCO Software Inc., Palo Alto, CA, USA).

## 4. Conclusions

The substitution of walnut oil cake (WOC) or hazelnut oil cake (HOC) for 10% of durum wheat semolina resulted in improved nutritional profile of pasta and, in the case of WOC, in notably higher antioxidant potential and total polyphenol content as compared to the standard product. The TDF content of HOCP allows for a high dietary fiber content nutritional claim. The culinary properties of the enriched pastas were typical of good quality pasta. The oil nut cakes made the pasta softer when uncooked and less elastic when cooked. Overall, fortifying durum wheat pasta with walnut or hazelnut oil cakes is a promising approach to enhance both nutritional value and functional properties. The increased bioactive compound content, particularly in WOC-enriched pasta, constitutes potential additional health benefits for consumers. Further research is recommended to optimize formulation and processing conditions to maximize the utilization of these nut oil cake by-products in pasta production.

## Figures and Tables

**Table 1:** Chemical composition of raw materials and products.

Sample	Component (g/100 g d.w.)						
	Protein	Fat	Ash	SDF	IDF	TDF	Available Carbohydrates
	**Raw Materials**						
Semolina	13.0 ^a^ ± 0.1	1.32 ^a^ ± 0.05	0.80 ^a^ ± 0.02	1.91 ^a^ ± 0.15	2.57 ^a^ ± 0.28	4.48 ^a^ ± 0.13	80.4 ^b^ ± 0.2
WOC	37.3 ^c^ ± 0.3	20.8 ^c^ ± 0.1	5.01 ^b^ ± 0.09	3.89 ^c^ ± 0.06	13.78 ^b^ ± 0.07	17.67 ^b^ ± 0.01	19.2 ^a^ ± 0.3
HOC	32.8 ^b^ ± 0.7	19.8 ^b^ ± 0.3	5.88 ^c^ ± 0.04	2.50 ^b^ ± 0.17	21.13 ^c^ ± 0.01	23.63 ^c^ ± 0.16	17.9 ^a^ ± 0.8
	**Products (Cooked and Dried)**						
SP	12.9 ^a^ ± 0.1	1.19 ^a^ ± 0.07	0.40 ^a^ ± 0.02	2.34 ^c^ ± 0.09	2.22 ^a^ ± 0.22	4.56 ^a^ ± 0.13	81.0 ^b^ ± 0.3
WOCP	15.8 ^c^ ± 0.1	3.64 ^b^ ± 0.16	0.66 ^b^ ± 0.02	1.26 ^a^ ± 0.12	3.91 ^b^ ± 0.12	5.16 ^a^ ± 0.23	74.8 ^a^ ± 0.4
HOCP	15.0 ^b^ ± 0.3	3.47 ^b^ ± 0.20	0.66 ^b^ ± 0.00	1.81 ^b^ ± 0.09	4.95 ^c^ ± 0.28	6.75 ^b^ ± 0.19	74.1 ^a^ ± 0.5

Explanations: SDF—soluble dietary fiber, IDF—insoluble dietary fiber, TDF—total dietary fiber. The values are reported as mean ± standard deviation. Means values in column, for raw materials or products, followed by the same superscript do not differ significantly at 0.05 significance level (Tukey test).

**Table 2:** Antioxidant activity and total phenolic content of uncooked and cooked pasta.

Sample	Antioxidant Activity			TPC (mg GAE/ 100 g d.w.)
	ABTS Assay (mmol TE/ 100 g d.w.)	DPPH Assay (mmol TE/ 100 g d.w.)	FRAP Assay (mmol TE/ 100 g d.w.)	
	**Uncooked**			
SP	0.73 ^a^ ± 0.02	0.04 ^a^ ± 0.01	0.01 ^a^ ± 0.00	80.95 ^b^ ± 3.65
WOCP	1.70 ^b^ ± 0.14	2.65 ^c^ ± 0.02	0.35 ^b^ ± 0.01	142.71 ^c^ ± 5.84
HOCP	0.64 ^a^ ± 0.03	0.31 ^b^ ± 0.04	0.03 ^a^ ± 0.00	65.79 ^a^ ± 2.89
	**Cooked**			
SP	0.63 ^a^ ± 0.07	0.20 ^a^ ± 0.11	0.02 ^a^ ± 0.00	88.85 ^a^ ± 1.40
WOCP	1.58 ^b^ ± 0.06	2.50 ^c^ ± 0.02	0.36 ^b^ ± 0.03	145.48 ^b^ ± 5.90
HOCP	0.73 ^a^ ± 0.01	0.45 ^b^ ± 0.03	0.05 ^a^ ± 0.00	89.40 ^a^ ± 4.38

The values are reported as mean ± standard deviation. Means values in column (separately for uncooked and cooked pasta) followed by the same superscript do not differ significantly at 0.05 significance level (Tukey test).

**Table 3:** Cooking properties of pasta.

Sample	Parameter			
	Optimal Cooking Time (min)	Water Absorption (g of water/100 g)	Swelling Index (g of water/g d.w.)	Cooking Loss (g d.w./100 g)
SP	11	124 ^a^ ± 8	1.37 ^a^ ± 0.01	4.52 ^a^ ± 0.27
WOCP	14	136 ^ab^ ± 2	1.47 ^b^ ± 0.03	5.79 ^b^ ± 0.14
HOCP	13	143 ^b^ ± 4	1.55 ^b^ ± 0.05	5.74 ^b^ ± 0.11

The values are reported as mean ± standard deviation. Means values in column followed by the same superscript do not differ significantly at 0.05 significance level (Tukey test).

**Table 4:** Color parameters of uncooked and cooked pasta.

Sample	Parameter					
	L*	a*	b*	L*	a*	b*
	**Uncooked**			**Cooked**		
SP	80.1 ^e^ ± 1.3	1.09 ^b^ ± 0.11	22.6 ^e^ ± 0.8	69.6 ^d^ ± 0.7	−1.49 ^a^ ± 0.11	14.1 ^b^ ± 0.6
WOCP	61.8 ^c^ ± 1.1	6.55 ^f^ ± 0.24	25.5 ^f^ ± 0.5	54.2 ^a^ ± 0.4	3.83 ^c^ ± 0.13	15.3 ^c^ ± 0.4
HOCP	61.2 ^c^ ± 1.7	6.10 ^e^ ± 0.13	18.5 ^d^ ± 0.6	56.7 ^b^ ± 0.3	4.14 ^d^ ± 0.12	10.6 ^a^ ± 0.3

The values are reported as mean ± standard deviation. Means values of given parameter followed by the same superscript do not differ significantly at 0.05 significance level (Tukey test).

**Table 5:** Texture parameters of pasta.

Sample	Test				
	3-Point Bending Test of Uncooked Pasta			Tensile Test of Cooked Pasta	
	Parameter				
	Breaking Force (N)	Displacement at Fracture (mm)	Breaking Force (N)	Elongation at Break (%)	Tensile Strength (kPa)
SP	5.21 ^c^ ± 0.15	0.21 ^b^ ± 0.02	0.452 ^b^ ± 0.030	129 ^b^ ± 25	29.0 ^b^ ± 1.8
WOCP	3.11 ^b^ ± 0.37	0.18 ^a^ ± 0.02	0.491 ^b^ ± 0.054	81 ^a^ ± 12	31.5 ^b^ ± 3.7
HOCP	2.50 ^a^ ± 0.11	0.17 ^a^ ± 0.01	0.360 ^a^ ± 0.025	63 ^a^ ± 5	23.1 ^a^ ± 1.5

The values are reported as mean ± standard deviation. Means values in column followed by the same superscript do not differ significantly at 0.05 significance level (Tukey test).

**Table 6:** RVA parameters of pasta powder.

Sample	Parameter		
	Pasting Temperature (°C)	Peak Viscosity (mPa∙s)	Final Viscosity (mPa∙s)
SP	86.1 ^a^ ± 0.3	705 ^b^ ± 48	2716 ^c^ ± 97
WOCP	88.0 ^b^ ± 0.5	531 ^a^ ± 21	1488 ^a^ ± 16
HOCP	89.1 ^c^ ± 0.6	506 ^a^ ± 18	1746 ^b^ ± 4

The values are reported as mean ± standard deviation. Means values in column followed by the same superscript do not differ significantly at 0.05 significance level (Tukey test).

## Data Availability

The raw data supporting the conclusions of this article will be made available by the authors on request.
